# Induced Superconducting Transition in Ultra-Thin Iron-Selenide Films by a Mg-Coating Process

**DOI:** 10.3390/ma14216383

**Published:** 2021-10-25

**Authors:** Zhiqiang Cao, Longqing Chen, Zhenxiang Cheng, Wenbin Qiu

**Affiliations:** 1 Instrumental Analysis Center, University of Shanghai for Science and Technology, Shanghai 200093, China; caozhiqiang@usst.edu.cn; 2 Key Laboratory of Radiation Physics and Technology of Ministry of Education, Institute of Nuclear Science and Technology, Sichuan University, Chengdu 610064, China; 3 Institute for Superconducting and Electronic Materials, Australian Institute for Innovative Materials, University of Wollongong, Squires Way, North Wollongong, NSW 2500, Australia; cheng@uow.edu.au

**Keywords:** thin film, iron-based superconductor, pulsed laser deposition, transmission electron microscopy

## Abstract

Binary Iron selenide (FeSe) thin films have been widely studied for years to unveil the high temperature superconductivity in iron-based superconductors. However, the origin of superconducting transition in this unconventional system is still under debate and worth deep investigations. In the present work, the transition from insulator to superconductor was achieved in non-superconducting FeSe ultrathin films (~8 nm) grown on calcium fluoride substrates via a simple in-situ Mg-coating by a pulsed laser deposition technique. The Mg-coated FeSe film with an optimized amount of Mg exhibited a superconducting critical temperature as 9.7 K and an upper critical field as 30.9 T. Through systematic characterizations on phase identification, carrier transport behavior and high-resolution microstructural features, the revival of superconductivity in FeSe ultrathin films is mostly attributed to the highly crystallized FeSe and extra electron doping received from external Mg-coating process. Although the top few FeSe layers are incorporated with Mg, most FeSe layers are intact and protected by a stable magnesium oxide layer. This work provides a new strategy to induce superconductivity in FeSe films with non-superconducting behavior, which might contribute to a more comprehensive understanding of iron-based superconductivity and the benefit to downstream applications such as magnetic resonance imaging, high-field magnets and electrical cables.

## 1. Introduction

Among the family of electrical materials, high-temperature superconducting material always attracts considerable attention not only considering its huge potential in high-efficiency electric transport and high-field magnets, but also due to the probable complement in condensed matter physics [1]. Iron-based superconductors [2,3,4,5] are considered one of the promising candidates that might unveil the mechanism of high-temperature superconductivity (HTS) since dramatic enhancement in critical temperature (*T*_c_) has been repeatedly achieved in binary iron selenide (FeSe) composite through a variety of strategies, including elemental substitution [6,7,8]/intercalation [9,10], pressurization [11,12], liquid-gate [13], and the substrate-induced heavy electron doping into FeSe unit cells [14,15,16]. Apparently, the HTS achieved in FeSe system reflects the exotic and unique properties in the unconventional Fe-based superconductors and is worth further investigation.

Although doping processes has been confirmed to dramatically affect the iron-based superconductivity, a comprehensive understanding with regards to the doping dependence of FeSe superconductivity is still being expected. Various approaches have been developed to investigate the doping effect onto FeSe superconductors and boosted *T*_c_ was realized in many ways. It is well accepted that the limited electron dopants transferred from STO substrate is not sufficient to drive the HTS in the second and beyond unit-cells [14,17,18,19]. A challenging issue has been raised which concerns how to supply more charge carriers. Miyata et al. [20] firstly reported the revival of HTS (~48 K) in multi-layer FeSe thin films by an in-situ post-deposition of K element. Extra electrons were introduced to FeSe thin films and the highest level of electron-doping in FeSe thin films was achieved. Shiogai et al. [21] proposed a novel method involving electrochemical etching and electric double-layer transistor to realize the accurate thickness controlling as well as the electron-doping into ultrathin FeSe thin films. Lei et al. [13] published the success of introducing massive electron carriers into FeSe thin flakes by a liquid-gating technique. A *T*_c_^onset^ of 48 K was induced in bulk FeSe and a Lifshitz transition was observed, indicating the dramatic change occurring in the Fermi energy as a result of the applied gate voltage. The deposition effect of Li element onto FeSe thin films was studied by Phan et al. [22] An enhanced *T*_c_ of 43 K was reported in a heavily-doped multilayer FeSe film. However, the lattice relaxation found in Li-doped FeSe films is in sharp contrast to the case of K-doping. Apart from the common scenarios of electron-doping, Sun et al. [23] transformed the superconducting phase of FeSe single-crystal from low-*T*_c_ to high-*T*_c_ by increasing pressure. They pointed out that the pressurization effect brings about inter-band spin fluctuation and reconstructs the Fermi surface of FeSe to a hole-dominated condition which is similar to the high-*T*_c_ FeAs superconductors and opposite to the heavily electron-doped FeSe thin films.

Inspired by the works of alkali metal (K, Li) deposition, we previously designed a similar post-deposition process for FeSe superconducting films [24] by coating typical alkaline-earth metal, Mg, which is much less active than K or Li so that a better chemical stability of coated FeSe composite film could be expected. A batch of pulsed laser deposition (PLD) prepared FeSe thin films with practical thickness (60 nm) were post-treated by an in-situ Mg-coating process and received an enhancement of *T*_c_ from 10.7 K to 13.4 K owing to the mild electron-doping effect [24]. Recently, a vanish of superconducting transition was found in ultrathin (~8 nm) PLD-prepared FeSe films grown on CaF_2_ substrates based on the investigation on thickness effect [25].

Therefore, herein, we propose a possible alternative to revive superconductivity in non-superconducting FeSe ultrathin films by introducing the external Mg-coating process. The pristine FeSe films with an average thickness as 8 nm has been certified to exhibit semiconducting behavior in *ρ*-T curve without superconducting transition. External electron-doping from the simple Mg-coating process and corresponding enhancement in superconducting performance of FeSe thin films are anticipiated if Mg-coating is proved to be a generic process to induce the superconductivity in FeSe.

## 2. Materials and Methods

Ultrathin FeSe films (~8 nm) were firstly grown on CaF_2_ (100) single crystal substrate (5 mm × 5 mm) via PLD (Nd: YAG, λ = 355 nm, 10 Hz, output ~2 W) with an average deposition rate as 1.7 nm/min. Right after the deposition of the FeSe layer, in-situ Mg-coating was performed at the same substrate temperature and the amount of Mg-coating was organized by controlling the deposition time as 3, 6.5, 10, 15, and 20 min. The chamber was evacuated to a vacuum state better than 5 × 10^−6^ Torr and the substrate temperature was fixed at 300 °C. The Mg-coated FeSe films were then cooled down to room temperature. All the samples are denoted in the order of Mg-amount as #UFM0 (pristine ultrathin FeSe), #UFM1 (3 min-Mg), #UFM2 (6.5 min-Mg), #UFM3 (10 min-Mg), #UFM4 (15 min-Mg), and #UFM5 (20 min-Mg), respectively.

Cu Kα X-ray diffraction (XRD, GBC MMA) *θ*-2*θ* scans were employed to identify phase structure. The electrical transport measurements including electrical conductivity and Hall measurement were carried out in a physical properties measurement system (PPMS 9 T, Quantum Design, San Diego, CA, USA). In order to investigate microstructural features and elemental information, an aberration-corrected scanning transmission electron microscope (STEM, ARM-200F, JEOL, Akishima, Japan) and an energy dispersive X-ray spectroscopy (EDS, Centurio SDD, JEOL, Akishima, Japan) were utilized. Further high-resolution chemical characterizations were conducted by electron energy loss spectroscopy (EELS) equipped on STEM at a spectral resolution of 0.05 eV. The electron-transparent lamellae for STEM observation was prepared using an in-situ lift-out technique in a focused-ion-beam (FIB, FEI Helios 600 NanoLab, Hillsboro, OR, USA) system.

## 3. Results and Discussions

**Figure 1:**
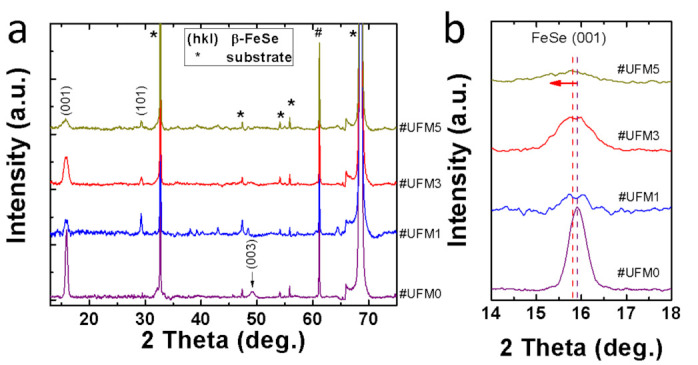
X-ray diffraction (XRD) θ-2θ patterns of #UFM0, #UFM1, #UFM3 and #UFM5. (hkl) signs represent the diffraction peaks of β-FeSe, while asterisk signs are originated from CaF_2_ substrate. (**a**) 2θ ranges from 12° to 75°; (**b**) A magnified interval near β-FeSe (001) peak from 14° to 18°. The peak position of #UFM0 and #UFM3 are marked by dashed lines, indicating a clear peak shift of FeSe (001) toward lower 2θ angle in Mg-coated #UFM3.

Typical XRD *θ*-2*θ* results for the pristine FeSe ultrathin film (#UFM0) and Mg-coated FeSe samples with a gradient of Mg amount (#UFM1, #UFM3, #UFM5) are illustrated in Figure 1a, ranging from 12° to 75°. Similar to our previous work [24], highly (00l) oriented FeSe texture based on PbO structure is observed in #UFM0. Once Mg was introduced, the diffraction peak indexed as FeSe (101) plane emerged in all Mg-coated samples, meanwhile the suppression of FeSe (00l) peaks indicated the reduction in the degree of orientation along *c*-axis upon Mg coating. As none of other phase was detected in all samples apart from those of FeSe and CaF_2_ substrate, we deduce that the mechanism in Mg-coated FeSe film might be different from the case of Mg-doped bulks [26] in which MgSe formed. From an enlarged interval near FeSe (001) peak (Figure 1b), a tiny peak shift toward lower angle in #UFM3 compared with pristine #UFM0 is noticed, implying the possible elongation of *c*-axis parameter after Mg-coating process. The phenomenon can be explained based on the interaction between Mg atoms and Fe-vacancies. During the Mg-coating process, Mg enters into FeSe lattice and occupies Fe-vacancies [24,27] in as-grown FeSe thin films, resulting in the elongation of FeSe lattice parameter. The filling effect of Fe-vacancies by Mg and the corresponding lattice elongation might leave impact on the superconducting behavior of Mg-coated FeSe films in consideration of their sensitivity to the subtle variation in lattice parameters [3]. Besides, residual stress is also possible to leave influence on superconducting performance with the compressed stress and tensile stress being beneficial and detrimental to the superconductivity of FeSe films in most cases [8,24,28,29,30]. Reflected by XRD results, the peak shift of FeSe almost saturates in #UFM3, in which the stress reaches to a maximum level and the highest *T*_c_ is obtained.

**Figure 2:**
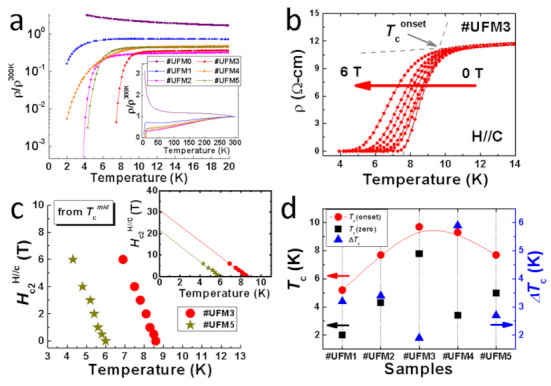
The results of resistivity behaviours of all samples in this work. (**a**) The temperature dependence of normalized resistivity *ρ*/*ρ*^300K^ up to 20 K. The *y*-axis is in logarithmic scale. Inset: temperature range up to 300 K. The *y*-axis is in linear scale; (**b**) *ρ*-*T* measurements for #UFM3 under external fields up to 6 T (parallel to c-axis); (**c**) Plots of *H*_c2_ as a function of *T*_c_^mid^ for #UFM3 and #UFM5. Inset: the linear extrapolations to T = 0 K; (**d**) The evolution of *T*_c_^onset^, *T*_c_^zero^, and *ΔT*_c_ with different amount of Mg-coating. The left *y*-axis refers to the *T*_c_^onset^ and *T*_c_^zero^, and the right *y*-axis stands for the Δ*T*_c_.

Figure 2a illustrates the temperature dependence (2–20 K) of normalized electrical resistivity *ρ*/*ρ*^300K^ for all six thin film samples in a logarithmic scale. The full range (up to room temperature) results are given in the inset of Figure 2a, demonstrating the metallic *ρ*-*T* behavior before *T*_c_ for the Mg-coated samples. Intriguingly, distinct superconducting transition is observed in all Mg-coated FeSe thin films, even for #UFM1 with a tiny amount of Mg addition. It is in sharp contrast to the pristine #UFM0 with a semiconducting behavior that is consistent with the case of other ultrathin Fe-chalcogenide thin films prepared by PLD and magnetron sputtering methods [21,25,31]. A similar phenomena of *T*_c_ revival in 8 nm non-superconducting FeSe thin films has been already observed in one of previous work in which a FeTe coating layer was introduced [32]. Here, for the case of *T*_c_ triggered in Mg-coated FeSe ultra-thin films, we prefer to deduce a possibility of a universal origin, which is attributed to a varied electronic orbital structure and a possibly lower electron occupancy [27] in Fe orbital near Mg/FeSe interfacial region [32]. The temperature dependence of resistivity under external field up to 6 T (parallel to *c*-axis) is shown in Figure 2b with the results of #UFM3 given as an example. The upper critical fields (*H*_c2_) of #UFM3 and #UFM5 are calculated depending on linear extrapolated *T*_c_^mid^ (the temperature at which resistivity drops by a half of the value of 14 K), with the *H*_c2_-*T*_c_^mid^ results plotted in Figure 2c. The estimated *H*_c2_ value for #UFM3 is about 30.9 T, exhibiting its good potential for the superconducting applications under high-field environments. The detailed specifications including *T*_c_^onset^, *T*_c_^zero^, and *ΔT*_c_ are provided in Figure 2d. With increasing the amount of Mg-coating, *T*_c_^onset^ raises from 5.2 K in #UFM1 to 9.7 K in #UFM3. The narrowest transition in #UFM3 (~1.9 K) suggests the good crystallinity of this sample, which can also be deduced based on the largest FeSe (001) diffraction peak intensity compared with that of other Mg-coated samples (as shown in Figure 1). Further increment in Mg addition gives rise to the degradation in *T*_c_^onset^ so that a dome-shaped dependence of *T*_c_^onset^ is obtained depending on the amount of Mg-coating. The transformation of resistivity behavior of FeSe layer from semiconducting to metallic should be related to the effect of extra electron-doping from external Mg-coating, which is believed to be one of the key factors that induce the superconductivity in ultrathin FeSe thin films.

**Figure 3:**
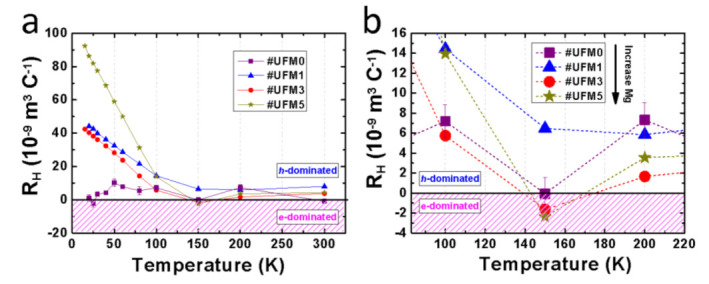
Hall coefficient *R*_H_ as a function of temperature for #UFM0, #UFM1, #UFM3 and #UFM5. (**a**) *R*_H_-*T* plots in a temperature range from 20 K to 300 K; (**b**) A magnified area from 90 K to 220 K, showing the phenomenon of sign reversal in #UFM3 and #UFM5.

It is well known that charge carrier concentration is one of the most crucial factors that determine the superconductivity in FeSe superconductors [17,20,33,34]. Considering that Mg element belongs to the group of alkaline-earth-metal in the periodic table, abundant electron carriers are supposed to be provided via Mg doping. Here, Hall measurements were performed to investigate the transport behavior of charge carriers. Figure 3a illustrates the temperature dependences of Hall coefficient (*R*_H_), which is defined as *R*_H_ = *ρ*_xy_⁄*B*, where *ρ*_xy_ stands for the Hall transverse resistivity and *B* is designated field under fixed temperatures ranging from 20 K to 300 K. From room temperature to 150 K, *R*_H_ of all samples was almost temperature independent. Below 100 K, positive *R*_H_ values were obtained in all the samples, indicating a hole-dominated situation of charge carriers. The correlation between *R*_H_ and carrier concentration (*n*) is deduced by

(1) $$R_{H}\;=\;\frac{E_{y}}{j_{x}B}\;=\;\frac{V_{H}t}{IB}\;=\;-\frac{1}{ne}$$

(*E*_y_—induced electric field, *j*—the current density of the carrier electrons, *B*—magnetic field, *V*_H_—Hall voltage, *t*—the thickness of the plate, *I*—the current across the plate length, *e*—elementary charge). As *n* is inversely proportional to *R*_H_, the increasing *R*_H_ in positive side at lower temperature region represents that *n* decreases with lowering temperature. The ultrathin #UFM0 without cap layer led to the fluctuation in *R*_H_ at low temperature region. Large absolute value of *R*_H_ was obtained in #UFM5 with the highest amount of Mg addition, suggesting a severe reduction in carrier concentration. It could be the result of the Mg oxide layer formed on the film surface due to the excessive Mg-coating. The transport property of #UFM5 was hence altered dramatically, which is consistent with the decaying superconducting performance in this sample (shown in Figure 2a). Thus, Hall’s measurements offer strong evidence for the degradation of the superconductivity in #UFM5 by revealing the collapse in carrier concentration.

The sign reversal of *R*_H_ usually suggests a dramatic change occurring to the transport property of charge carriers. An enlarged view of *R*_H_-*T* ranging from 90 K to 220 K is displayed in Figure 3b. We noted that the sign of *R*_H_ of #UFM3 and #UFM5 switched twice during the cooling process near 150 K, while that of the other two samples stayed on the positive side. Similar phenomenon of *R*_H_ sign reversal has been reported by Sun et al. [35] in their FeSe and FeSe_0.86_S_0.14_ single crystals. Obviously, more contribution to the transport performance is provided by electron-type carriers in #UFM3 and #UFM5 near 150 K, evidencing the effective electron-doping into ultrathin FeSe thin films by Mg-coating. However, the external electron-doping was not high enough to completely overturn the hole-dominated condition in pristine FeSe or Mg-coated FeSe composite films. It explains why the *T*_c_ in Mg-coated FeSe is not comparable to the case of K-coated multilayer FeSe [20,36,37] or liquid-gating [13] treated FeSe thin flakes which exhibited electron-domination even at low-temperature region. Further increment in Mg addition results in no trace of more electron contribution, reflecting the limited capacity of electron-doping by external Mg-coating. Therefore, we consider the sign reversal of *R*_H_ as a signature of higher *T*_c_ value in this work induced by electron-doping.

**Figure 4:**
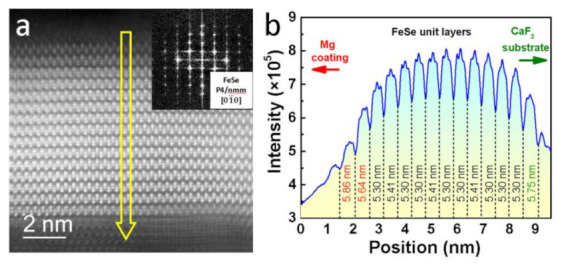
Scanning transmission electron microscopy (STEM) analyses for #UFM3 sample focusing on the cross-sectional region covering the entire FeSe layer. (**a**) Dark-field image. The inset provides a fast Fourier transform (FFT) pattern for FeSe core layers (zone axis $\left[0\bar{1}0\right]$); (**b**) A linear profile based on the contrast fluctuation of the arrow indicated in (**a**). The left/right side refers to the region of Mg-coating/CaF_2_ substrate. The width values represent the *c*-axis lattice parameters of each FeSe unit layer.

Figure 4 shows the STEM analyses on #UFM3 sample. The cross-sectional region covering the entire FeSe layer is clearly displayed in Figure 4a. About 15 FeSe unit layers (~8 nm in all) are clearly illustrated with the top and bottom layers being adjacent to Mg-Coating and CaSe [25] interlayer, respectively. The FeSe unit layers are fully intact without any obvious disorder or defect, indicating the good quality of the pristine ultrathin FeSe layer prepared on CaF_2_ substrate and the absence of large-scale interaction between FeSe layer and Mg-coating. The corresponding fast Fourier transform (FFT) pattern is given in the inset of Figure 4a, demonstrating a typical P4/nmm space group from $\left[0\bar{1}0\right]$ zone axis. A linear profile based on contrast fluctuation was conducted throughout the entire FeSe layer with the results of position dependent intensity shown in Figure 4b. Considering of the layered structure of FeSe and the zone axis of $\left[0\bar{1}0\right]$, the widths in the position axis between two neighboring valleys indicated the lattice parameter of each FeSe layer along *c*-axis and were individually measured. We noticed that three parts could be divided from the width distribution: the part near Mg-coating, the part of core FeSe layers, and the part near CaSe interlayer. For the case of the bottom FeSe layer with a *c*-axis lattice parameter as 5.75 nm, the expanded value derives from the CaSe interlayer [25] with a lattice parameter (*c* = 5.92 Å) much larger than that of FeSe. The top few FeSe layers possess the *c*-axis lattice parameters that gradually increase toward Mg-coating region (5.30 nm → 5.64 nm → 5.86 nm) owing to the filling effect of Fe-vacancies, which is consistent with XRD results.

**Figure 5:**
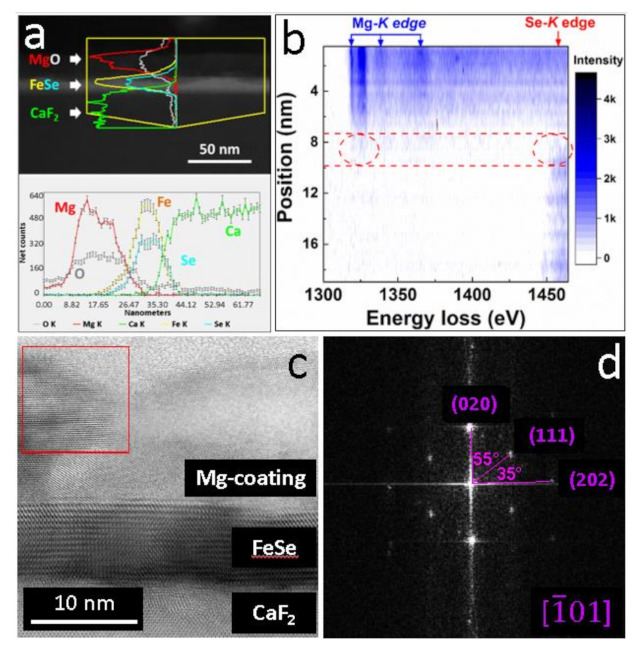
The STEM-related characterizations for #UFM3 from a cross-sectional view. (**a**) EDS linear-scanning showing the distribution of Mg, O, Fe, Se and Ca elements; (**b**) The EELS contour image illustrating the evolution of Mg-*K* and Se-*K* edges. The region in which Mg and Se coexist is highlighted between dashed lines; (**c**) A bright-field STEM image captured from the zone axis of $\left[\bar{1}01\right]$; (**d**) The FFT pattern of the polycrystalline region in the square in (**c**).

In order to further investigate the properties of Mg-coating and its interaction with FeSe films, detailed STEM characterizations were performed with the results given in Figure 5. The EDS linear-scanning results for Mg, O, Fe, Se and Ca elements are illustrated in Figure 5a. According to the diverse appearance of the net-count curves, the distribution of different elements can be estimated. Apparently, most of the Mg dwelled above FeSe region together with a big amount of O. The inter-diffusion of Mg into FeSe layer degraded rapidly and almost no Mg element was detected deep inside core FeSe layers. Figure 5b is an EELS contour containing the position-dependent energy loss spectra for Mg-*K* and Se-*K* edges. Darker contrast indicates higher intensity of the characteristic edges. EELS results are very sensitive in distinguishing different elements because that the mode of orbital excitation in every element is unique [32,38,39]. In addition to the Mg coating on the top of FeSe layer which was verified by EDS-mappings, a region with the presence of both Mg-*K* and Se-*K* edges was distinguished near the Mg/FeSe interface. Therefore, two conclusions can be made based on the STEM-EDS and EELS results: (1) Most of the Mg dwelled above FeSe layers and almost no Mg element diffused into core FeSe layers. It excluded the possibility of Mg-intercalation in this work. (2) Mg diffused into the top few layers of FeSe film, which was probably responsible for the evolution of the electrical transport and superconducting properties. A bright-field STEM image captured from the zone axis of $\left[\bar{1}01\right]$ is displayed in Figure 5c. The FeSe layers dwell between Mg-coating and CaF_2_ regions. Above FeSe layer, the region of Mg-coating contains both polycrystalline lattices and amorphous phases. FFT was conducted onto a 10 nm × 10 nm area in Mg-coating region (square in Figure 5c) with the results shown in Figure 5d. The corresponding patterns is well matched with the cubic magnesium oxide (MgO, *a* = 4.2170 Å, space group: $Fm\bar{3}m$). Thus, we deduce that the excess Mg-coating on the surface of FeSe layer finally got oxidized and transformed into polycrystalline MgO. The high stability of MgO is considered to offer protection to the beneath ultrathin FeSe films.

## 4. Conclusions

In summary, we carried out a simple Mg-coating process onto non-superconducting FeSe ultrathin (~8 nm) films and obtained a positive result of superconducting transition (*T*_c_ = 9.7 K) as well as evidence of extra electron-doping from external Mg-coating. Once Mg-coating was introduced into highly crystallized FeSe (00l) grown on CaF_2_ substrate, an FeSe (101) orientation emerged and lattice was slightly elongated along *c*-axis. According to the Hall measurements, a double sign reversal of *R*_H_ near 150 K was detected in heavily Mg-coated FeSe films and verified the effective electron-doping which induced the higher *T*_c_ values in these samples. High-resolution STEM, EDS, and EELS characterizations revealed that most FeSe layers were intact and defectless while only top few FeSe layers were diffused by Mg, resulting in the expansion in lattice parameter and variation in electrical transport properties. The excessive Mg-coating above FeSe region converted to MgO and provided extra protection to the beneath FeSe ultrathin films. This work is the first attempt to achieve superconducting transition non-superconducting FeSe ultrathin films via external Mg-coating process. Based on the results, a unified mechanism is deduced that the introduction of the elements from the family of alkali/alkaline-earth metals will cause a generic consequence of electron-doping, which is one of the prerequisites that might trigger HTS in FeSe system. The clarification might also profit the downstream applications of iron-based superconducting thin films, including magnetic resonance imaging, high-field magnets, electrical cables, etc.
